# Total Humeral Endoprosthetic Replacement following Excision of Malignant Bone Tumors

**DOI:** 10.1155/2016/6318060

**Published:** 2016-02-21

**Authors:** Suhel Kotwal, Bryan Moon, Patrick Lin, Robert Satcher, Valerae Lewis

**Affiliations:** ^1^Orthopedic Surgery, University of Missouri-Kansas City, Kansas City, MO 64018, USA; ^2^Orthopaedic Oncology, Division of Surgery, The University of Texas MD Anderson Cancer Center, Houston, TX 77030, USA

## Abstract

Humerus is a common site for malignant tumors. Advances in adjuvant therapies and reconstructive methods provide salvage of the upper limb with improved outcomes. Reports of limb salvage with total humeral replacement in extensive humeral tumors are sparse. We undertook a retrospective study of 20 patients who underwent total humeral endoprosthetic replacement as limb salvage following excision of extensile malignant tumor from 1990 to 2011. With an average followup of 42.9, functional and oncological outcomes were analyzed. Ten patients were still alive at the time of review. Mean estimated blood loss was 1131 mL and duration of surgery was 314 minutes. Deep infection was encountered in one patient requiring debridement while mechanical loosening of ulnar component was identified in one patient. Subluxation of prosthetic humeral head was noted in 3 patients. Mean active shoulder abduction was 12.5° and active flexion was 15°. Incompetence of abduction mechanism was the major determinant of poor active functional outcome. Mean elbow flexion was 103.5° with 30.5° flexion contracture in 10 patients with good and useful hand function. Average MSTS score was 71.5%. Total humeral replacement is a reliable treatment option in restoring mechanical stability and reasonable functional results without compromising patient survival, with low complication rate.

## 1. Introduction

The humerus is commonly affected by primary and secondary malignant bone tumors that may require radical surgical excision [1]. Compared to ablative surgery, limb salvage offers cost-effective and improved functional outcomes with no difference in the overall patient survival [2–4]. In combination with neoadjuvant or adjuvant therapy and complimented by technological advances in surgical techniques, radiographic data, and implant engineering, limb salvage has been established as the mainstay of the treatment [5–7].

Following partial humeral resection, the options for reconstruction include autograft, osteoarticular allograft, alloprosthetic composite, and segmental custom-made or modular endoprosthetic replacement [8–12]. Reconstruction with allografts results in improved relative active function of the shoulder but has associated drawbacks of fracture, nonunion, and infection [13, 14]. Although endoprosthetic reconstructions also have potential complications including, infection, aseptic loosening, dislocation, and mechanical failure, they are a preferred choice as they impart immediate stability and weight bearing to the extremity, eliminating the complications of allografts [15–18]. However, cases which require complete excision of the humerus present a unique reconstructive challenge. The use of cadaveric osteoarticular allograft or alloprosthetic composite to replace the entire length of the humerus is unfeasible as this requires replacement of both glenohumeral and elbow joints and there is absence of a healing interface or source of vascularity to the graft. Thus endoprosthetic reconstruction remains the procedure of choice for limb salvage for extensive malignant bone lesions that require excision of the entire humerus [19, 20].

To date, reports on the functional and oncological outcome of patients who have undergone total humerus endoprosthetic reconstruction for malignant bone tumors are sparse in literature [19, 20]. We describe our experience of employing this procedure for the past two decades using both custom-made and modular total humeral endoprosthetic implants.

## 2. Material and Methods

**Figure 1:**
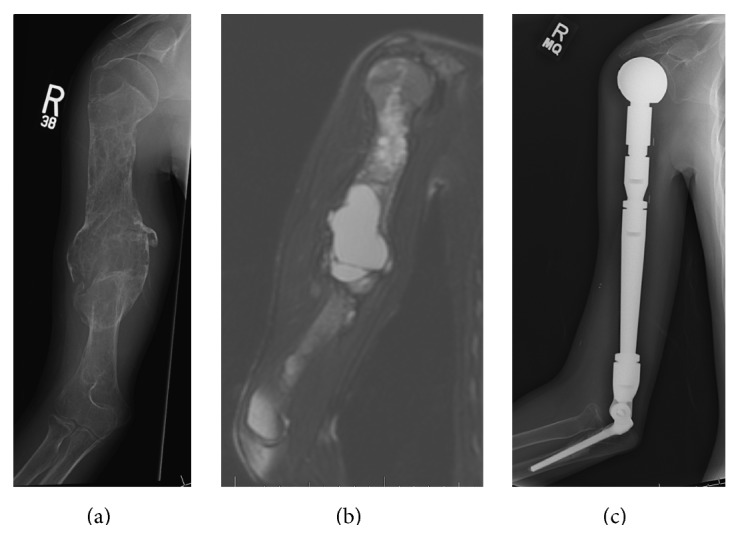
16-year-old male with telangiectatic osteosarcoma of the humeral shaft, treated with total humeral resection and endoprosthetic construction.

Between January 1990 and December 2011, 20 patients underwent total humeral endoprosthetic reconstruction at our institution (Figures 1(a)–1(c)). All procedures were carried out by surgeons who were fellowship-trained in musculoskeletal oncology, at a single tertiary referral bone tumor center. Following Institutional Board Review approval, data was collected through retrospective analysis of medical records, imaging studies, and questionnaires structured for measurement of patient-derived outcome. Of the 20 patients, 11 were treated for primary humeral sarcoma and 9 for metastatic disease of a known distant carcinoma (Table 1). There were 10 males and 10 females, with a mean age of 40.9 years (range, 4–70). All patients underwent standard preoperative staging, including plain radiographs, Magnetic Resonance Imaging (MRI) of the humerus, Computed Tomography (CT) scan of the chest, and bone scintigraphy. The primary disease was staged according to the Musculoskeletal Tumor Society (MSTS) scoring system [21]. A total of 13 core needle and 8 open biopsies, including frozen section examination during surgery, provided the histological diagnoses in these patients. All patients underwent appropriate neoadjuvant and adjuvant chemo- and radiotherapy according to their respective treatment protocols.

### 2.1. Prosthesis

The total humeral endoprostheses were available to us in modular and custom-made designs. We utilized the custom design in the pediatric subgroup of patients in combination with a growing segment for potential lengthening. The modular prostheses (Stryker Orthopedic Inc., Marwah, NJ & Biomet Orthopedic Inc., Warsaw, IN) comprises of a monoblock, cobalt chrome humeral head component with titanium diaphyseal segments of sequentially increasing lengths, with a fixed hinge to replace the elbow joint. The modular segments are connected with axially impacted trunnion and bore type Morse tapers. The ulnar component comprises of a straight tapered, grit-blasted, cemented stem with varying length options. We routinely utilized short stems (55 mm) in our 9 initial cases but revised our choice of stem length to 100 mm to offer improved cemented fixation in the later part of our series. Detailed measurement of the contralateral humerus during preoperative planning determined the length of the prosthesis along with size of the humeral head, especially in cases where custom prostheses were planned to be used. Provisions for reattachment of the rotator cuff and deltoid tendons to the prostheses were made with surface areas of hydroxyapatite (HA) or plasma porous coating at strategic locations. A standard retroversion of 40° of the humeral head with respect to the diaphysis was prefixed before implantation.

### 2.2. Surgical Technique

Patient is positioned in either supine, semireclining, or beach-chair position with head and endotracheal tube well secured. An extensile approach to the humerus is utilized for this procedure comprising a combination of the standard deltopectoral approach to the shoulder, anterolateral approach to the humerus, and anterior approach to the elbow [22, 23]. The incision is modified to incorporate excision of the previous biopsy tract whenever required. Unless preoperative decision of an extra-articular excision was made, the rotator cuff, biceps, and deltoid tendons were preserved and tagged, without compromising adequate tumor clearance. Likewise, the neurovascular structures including the axillary, radial, median, and ulnar nerves along with brachial artery are identified, isolated, and preserved. The humeral head is dislocated and the entire bone along with the tumor is delivered after transection of the collateral ligaments of the elbow. Care is taken to preserve the triceps tendon continuity with the olecranon process wherever possible. The proximal ulna is reamed to receive the cemented stem component of the hinge. The prosthesis is then assembled on the back table and implanted by assembling the hinge mechanism and reducing the humeral head. Following implantation and seating of the prosthesis, all attempts are made to establish a stable glenohumeral joint with meticulous repair and reattachment of the rotator cuff, deltoid, and short head of biceps tendons, using fiber-wire suture or mersilene tape. Intravenous antibiotics are continued for three days following operation. In the initial postoperative phase, we place the arm in a flexion-abduction orthotic for a total period of 3 weeks without allowing any motion involving the shoulder although active elbow and hand motion are encouraged. Passive, assisted abduction and flexion of the shoulder are encouraged thereafter with the brace discontinued and replaced by an antigravity sling. Range of motion of shoulder and elbow joints, both active and passive, was measured using a goniometer. Each patient received plain radiographic exam of the prosthesis at each office visit to determine any evidence of dislocation, subluxation, aseptic loosening, mechanical failure, or fracture.

The Musculoskeletal Tumor Society (MSTS) score, which is a clinician scored system for the extremities following reconstructive procedures after skeletal excision, was utilized to assess the functional outcome at each office visit. The MSTS scoring system comprises a six-item scale that has numerical values (0–5) assigned to each of the six categories of pain, function, emotional acceptance, use of supports, walking ability, and gait, to produce a score ranging from 0 to 30. These values are added, and the functional score is presented as a percentage of the maximum possible score [24].

### 2.3. Statistical Analysis

Kaplan-Meier survival curves for both implant and patient were used to compare rates of survival. Survival of the implant was analyzed with an endpoint identified as reoperation. Patients were censored for statistical analysis (observation stopped before the event occurred) if the failure had not occurred at the time the patient was last assessed. Patient times of death were also censored at the time of implant failure in cases where the implant failed before death.

## 3. Results

**Figure 2:**
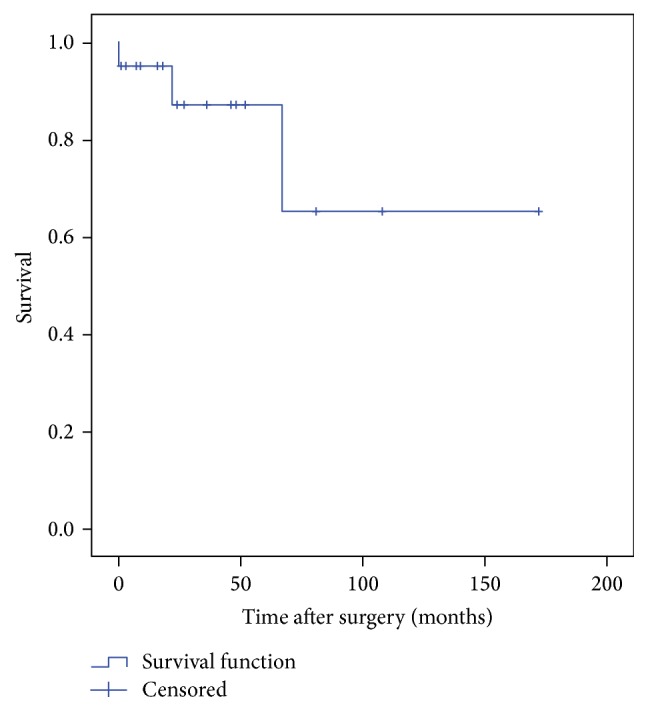
Implant survival.

**Figure 3:**
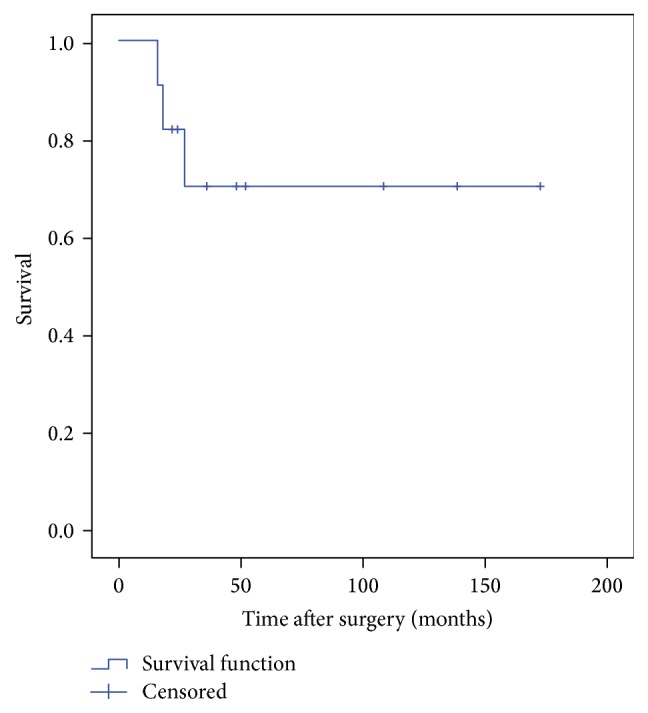
Patient survival.

The mean follow-up was 42.9 months (range, 1–172 mts) for all patients. Eleven patients were alive at the time of review while 9 succumbed to metastatic disease. Implant survival was 87.1% at 5-year and 65.3% 10-year marks with the endpoint being revision surgery for mechanical failure (Figure 2). Patient survival was 70.1% at 10 years (Figure 3).

Of the 11 patients with primary bone sarcomas, 8 had a common diagnosis of osteosarcoma, while each was diagnosed with chondrosarcoma, Ewing's sarcoma, and a histologically unclassified sarcoma. Renal cell carcinoma was most common diagnosis in the metastatic group with 6 patients, while the remaining three were diagnosed with breast carcinoma, thyroid carcinoma, and metastatic alveolar soft part sarcoma. A total of five patients presented to us following a previous operation with four (2 patients each in primary and metastatic group) who had intramedullary nailing, and one patient who had a proximal humeral resection and endoprosthetic reconstruction (Table 2).

Five patients, all with a diagnosis of primary sarcoma, required an extra-articular glenohumeral resection with a complete excision of rotator cuff tendons and glenoid process in an attempt to achieve adequate bone and soft tissue tumor margin clearance. In these cases, the proximal end of the prostheses, minus the unipolar head, was suspended with stainless steel cables or mersilene tapes from the acromion process through drill holes. Radical excision of soft tissues in 6 cases required the services of plastic surgery colleagues who employed the ipsilateral rotational latissimus dorsi myocutaneous flap (LDMF) for adequate prostheses and wound coverage. Mean surgical time was 314 minutes (range, 57–653) with an average estimated blood loss of 1131 mL (range, 100–6050 mL) requiring 3.5 units of transfused blood during and after surgery (Table 3). A traumatic rent in the brachial artery was encountered in one case, which required direct full-thickness, vessel wall repair with nonabsorbable suture. Two patients were diagnosed with radial nerve paralysis in the immediate postoperative period, which did not show any signs of recovery till the last available follow-up. Total complication rate in our series was 25%. Patients spent an average of 8.1 days (range, 3–31 days) in the hospital and later were discharged from the hospital with home health services.

Wide bone and soft tissue margins were obtained during surgery, as confirmed with intraoperative frozen section examination. Local recurrence was noted in one patient (5%) with metastatic renal carcinoma that required three different procedures for successful resection followed by radiation therapy. This patient also developed a deep, delayed prosthetic wound infection 6 weeks following surgery, which required irrigation and debridement and intravenous antibiotic therapy with successful eradication. One patient was noted to have aseptic loosening of the ulna stem at his 9-year follow-up but remained asymptomatic without any pain or loss of function; hence a decision was made to defer revision surgery for the stem. There was one case of superficial surgical site infection with wound dehiscence that required debridement, wound revision, and closure with subsequent healing.

### 3.1. Three Patients Required Revision Surgery for Implant Related Mechanical Failure

**Figure 4:**
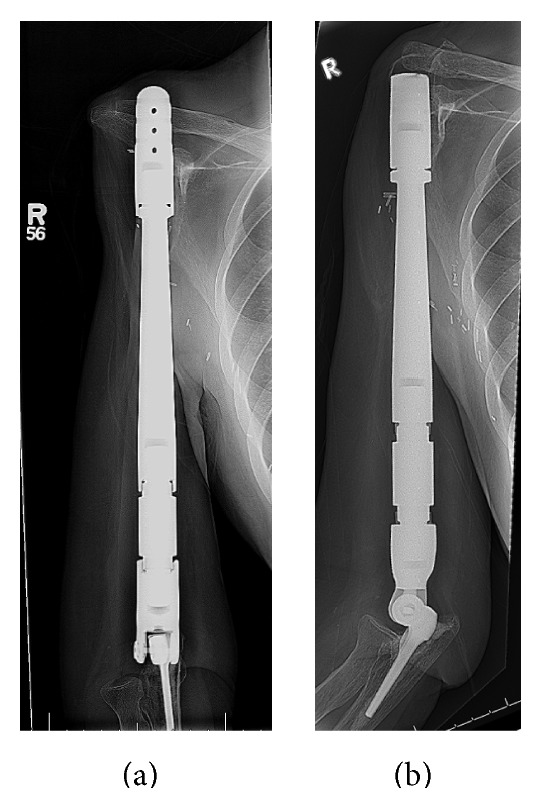


*Case  1.* 32-year-old lady presented with a pathological fracture of the right humerus, treated with interlocked, intramedullary nail at an outside facility with significant pain and nonunion. Biopsy at our institution revealed the diagnosis of high-grade fibroblastic osteosarcoma. She underwent resection of the entire right humerus with extra-articular (modified Tikhoff-Linberg) glenohumeral excision. At her 5th year follow-up, she presented with significant complaint of pain at the shoulder joint, which worsened with motion. Disuse atrophy of the deltoid muscle gave her an ungainly prominence of the proximal end cap of prosthesis. She was treated with revision surgery in the form of reduction of length of the prosthesis by removal of the end cap and reattachment through drill holes, to the scapula with mersilene tape. She remained relatively pain-free till her 12th annual follow-up where she had identical symptoms of pain with motion and unsightly prominence of the prosthesis. She underwent her second revision surgery, which involved reduction of the prosthetic length by an additional 2 cm. This resulted in satisfactory pain control that was well maintained till her latest follow-up (Figures 4(a) and 4(b)).

**Figure 5:**
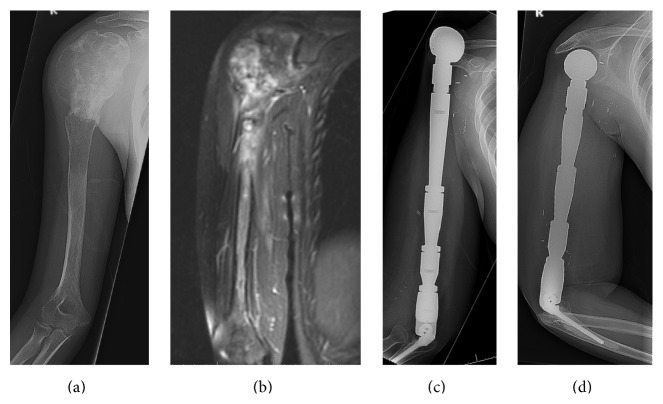


*Case  2*. 23-year-old lady presented with high-grade chondroblastic osteosarcoma of her right humerus. Her history was complicated by a pathological fracture of the humeral shaft, treated nonoperatively by her own choice, in favor of holistic therapy. At presentation, she had extensive involvement of the humerus that required a total humeral resection and endoprosthetic replacement. At her 22nd postoperative month office visit, she presented with significant anterior and proximal subluxation of the prosthetic head with painful impingement. She underwent a revision procedure in the form of reduction in the diameter of both the humeral head and length of diaphyseal segment along with reattachment to the glenoid and acromion process using a Dacron mesh wrapped around the unipolar humeral head (Figures 5(a)–5(c)).

*Case  3*. 65-year-old man with metastatic renal cell carcinoma to the right proximal humerus, treated with intramedullary nail at an outside facility, presented to us with massive tumor recurrence and pathological fracture. He underwent total humeral excision and endoprosthetic replacement but incurred dislocation of the humeral head in the immediate postoperative period. He was revised on day 2 of his primary surgery in the form of relocation of the prosthesis with meticulous repair of his rotator cuff, deltoid, and short head of biceps tendons, with nonabsorbable suture.

### 3.2. Functional Outcome

Overall the mean MSTS functional outcome score for all 20 patients at the time of their respective latest available follow-ups was 71.5%. Of the 11 surviving patients available for evaluation, the mean score was 77.6%. All patients had predominantly passive rather than active motion of the shoulder and none could actively elevate their arm against gravity to face level in either flexion or abduction. With the elimination of the scapulothoracic compensation, the mean active flexion and abduction were 12.5°. All patients recovered satisfactory range of motion in their elbow with a mean flexion of 103.5° (range, 0–130°) and a residual flexion contracture of 30.5° (range, 5–60°), in 10 patients. The majority of patients had full hand and wrist function. Two patients required the use of drop-wrist orthotic due to radial nerve dysfunction.

## 4. Discussion

Limb salvage has replaced amputation as the standard of care for the treatment of primary and secondary malignant tumors of long bones including humerus, largely owing to the improvements in chemotherapy [2, 4, 6, 7, 25–27]. Overall patient survival following limb salvage is the same as amputation, albeit at a higher rate for local recurrence [6, 7]. Alloprosthetic composite, osteoarticular allograft, and endoprosthetic replacement are the commonly preferred reconstructive options following partial resection of long bones, including humerus. However, large malignant tumors of humerus may present with extensive diaphyseal involvement or skip metastases that require major excision, rendering inadequate residual host bone, incapable of secure allograft or endoprosthetic stem fixation [19, 20]. Our study suggests that, with allograft options impracticable in such cases, total humeral endoprosthetic replacement with articulation at glenoid and ulna offers a safe, feasible, and reliable option. Among the major advantages, high level of emotional acceptance, residual useful functional ability of the elbow and hand are achieved without compromising tumor clearance or high recurrence and complication rates [19, 20, 28, 29]. This imparts immediate stability to the upper extremity without concerns of nonunion or delayed union, especially in cases which proceed to radiation and chemotherapy postoperatively. The significance of our study, which is the largest series to date, is that it examines the various indications, surgical technique, prosthetic design, perioperative parameters, complications, and function following this rare type of reconstruction.

A common shortcoming of this procedure is failure to achieve useful and active antigravity motion, predominantly due to incompetency of the intrinsic stabilizers of the glenohumeral joint, including the rotator cuff tendons [19, 30]. Although attempts are made at reattachment of the tendons on rough, receptive surfaces of the prostheses and immobilization of the shoulder in abduction to relax the repair while healing, the result is largely inadequate with our current techniques. This renders unopposed contraction of deltoid leading to superior subluxation of the prosthetic head. This migration of the unipolar humeral head creates an unstable glenohumeral fulcrum and loss of mechanical advantage, which subsequently results in poor active motion of the glenohumeral joint in the immediate postoperative phase. It is imperative to counsel the patient and his/her support group about the possibility of inadequate active motion and the need to reeducate and modify shoulder dynamics for a protracted course. Motivated patients constantly train and reinvent their respective shoulder girdles to employ scapula-thoracic compensation combined with adaptation of lifestyle, thereby enabling them to carry out common activities of daily living. Useful elbow and hand function contributes towards this goal to a large extent. The majority of our patients (65%) were found to have radiographic evidence of superior migration of the head, which was clinically asymptomatic and did not require intervention. However, painful subacromial impingement in two patients required surgical mediation, in form of shortening of the prosthetic diaphyseal segment, reduction in the humeral head size, and reattachment of rotator cuff and deltoid tendons. Efforts should be made to examine clinical and radiographic signs of glenohumeral incongruence at each clinic visit.

Reports of total humerus endoprosthetic replacements have been sparse in literature. Natarajan et al. reviewed 11 patients with malignant tumors of the humerus who underwent radical excision and reconstruction with custom-made total humerus endoprostheses [19]. The authors described good to excellent one and five-year cumulative survival rates at 90.9% and 77.9%, respectively. Patients in their series had minimal active shoulder motion, but relatively good elbow and hand function with an average MSTS score of 80%, comparable to our study. In another study, Funovics et al. reported 11 cases reconstructed with total humeral replacement following tumor excision with a final MSTS score of 70% and a deep infection in 3 cases [20]. However, shoulder function was not specifically evaluated in this series. Several investigators have described the results of their experience with proximal and distal humeral endoprosthetic replacements through discrete reports. Cannon et al. examined the functional outcome of 83 proximal humeral replacements following tumor resection [30]. At a mean follow-up of 30 months the MSTS score was 63%, although the active abduction and flexion of 42° were better than our current series. This difference may be due to exclusion of extra-articular excisions in this study, while 5 patients in our series underwent modified Tikhoff-Linberg procedure with excision of rotator cuff tendons. Twenty-two of 83 patients were reported to have proximal migration of humeral head in this study, while none required revision for symptomatic impingement. Use of synthetic nylon or Prolene mesh wrapped around the humeral head for improved anchoring of muscles has been attempted with improved motion and fewer dislocations [31]. Tang et al. reported an MSTS score of 23.9 points following distal humeral resection and prosthetic replacement in 25 patients [32]. Local recurrence of 24% in this series is higher compared to ours (5%) but complication rates and range of elbow flexion, with an arc of over 100°, were similar.

Our case series has several limitations including small sample size, short follow-up, single institution experience, and lack of dedicated scoring system for separate evaluation of individual joints including shoulder and elbow. However, these drawbacks are inherent to such retrospective studies of rare oncological procedures performed for malignant tumors. Total humeral endoprosthetic replacement is a valuable tool that offers the opportunity of limb salvage following extensive and total excision of the humerus. It carries a low complication rate, improved emotional acceptability, and enhanced function for the operated extremity, with preservation of elbow and hand function. Advances in soft tissue anchoring techniques over metal surfaces with use of synthetic mesh may result in improved active motion and fewer dislocations. With careful selection and preoperative counseling, with respect to limited active shoulder motion, this reconstructive modality is a valuable tool in the treatment of rare extensive malignant tumors of the humerus.

## Figures and Tables

**Table 1:** Demographics and surgical details.

Case	Gender	Age (yrs)	Diagnosis	Previous treatment	Plastic surgery	Ulna stem	EBL (mL)	DOS (min)	LOS (days)
*Primary*									
1	M	67	Unclassified	Cx	IR + LDMF	Long	850	347	13
2	F	11	OS-osteoblastic	Cx	ER + LDMF	Short	800	314	7
3	M	38	CS	Cx	ER + LDMF	Long	600	325	8
4	F	12	OS	Cx	ER + LDMF	Short	1075	301	5
5	F	70	OS-high-grade surface	Cx, IM nail	IR	Short	500	345	5
6	F	13	ES	Cx	IR	Long	1150	165	4
7	F	32	OS-fibroblastic	Cx, IM nail	ER + LDMF	Short	400	180	8
8	F	4	OS-osteoblastic	Cx	ER	Short	100	57	4
9	M	16	OS-telangiectatic	Cx	IR	Long	550	362	3
10	M	9	OS-high-grade surface	Cx	IR	Short	250	339	8
11	F	24	OS chondroblastic	Cx	IR + LDMF	Long	1000	469	9

*Metastatic*									
12	F	46	Breast		IR	Short	4000	210	10
13	M	57	Thyroid		IR	Long	1800	271	7
14	M	69	RCC		IR	Long	1000	266	7
15	M	59	RCC	IM nail	IR	Short	350	182	3
16	M	62	RCC	IM nail	IR	Long	400	653	9
17	M	65	RCC		IR	Short	6050	351	5
18	M	66	RCC		IR	Long	900	394	2
19	F	36	ASPS		IR	Long	600	513	31
20	F	62	RCC	Endoprosthesis (prox. humeral)	IR	Long	250	237	14

**Table 2:** Outcome.

Case	Complications	Additional treatment	Follow-up (mos)	MSTS score (%)	ROM shoulder (degrees)		ROM elbow (degrees)		Recurrence	Status
					Abduction	Flexion	Flexion	Extension		
*Primary*										
1	Subluxation		18	77	5	5	130	0	N	DOD
2			16	77	0	0	100	20	N	DOD
3			52	95	0	0	90	20	N	NED
4			36	63	30	30	100	30	N	DOD
5	Subluxation, loosening		108	73	5	5	80	0	N	NED
6	Subluxation		172	63	0	0	100	0	N	NED
7	Dislocation	Revision (67 mos)	138	48	10	10	130	0	N	NED
8			27	100	110	110	100	40	N	DOD
9			48	73	40	40	130	0	N	NED
10			24	80	NA	NA	90	0	N	NED
11	Subluxation	Revision (22 mos)	22	66	10	10	110	5	N	NED

*Metastatic*										
12	Wound dehiscence	Wound revision (35 d)	3	53	0	0	100	20	N	DOD
13			9	77	5	5	100	0	N	AWD
14			1	73	NA	NA	NA	NA	N	DOD
15	RNP, infection, and subluxation	Irrigation & debridement	46	40	NA	NA	100	30	Y	DOD
16			81	83	0	0	120	20	N	AWD
17	RNP, subluxation		18	67	0	0	110	10	N	DOD
18	Dislocation	Revision (2 days)	7	53	0	0	120	60	N	DOD
19			7	82	0	0	30	0	N	DOD
20	Subluxation		24	87	10	10	110	20	N	AWD

**Table 3:** Perioperative parameters.

Case	EBL (mL)	DOS (min)	LOS (days)
*Primary*			
1	850	347	13
2	800	314	7
3	600	325	8
4	1075	301	5
5	500	345	5
6	1150	165	4
7	400	180	8
8	100	57	4
9	550	362	3
10	250	339	8
11	1000	469	9

*Metastatic*			
12	4000	210	10
13	1800	271	7
14	1000	266	7
15	350	182	3
16	400	653	9
17	6050	351	5
18	900	394	2
19	600	513	31
20	250	237	14
